# Langerhans cells and cDC1s play redundant roles in mRNA-LNP induced protective anti-influenza and anti-SARS-CoV-2 immune responses

**DOI:** 10.1371/journal.ppat.1010255

**Published:** 2022-01-24

**Authors:** Sonia Ndeupen, Aurélie Bouteau, Christopher Herbst, Zhen Qin, Sonya Jacobsen, Nicholas E. Powers, Zachary Hutchins, Drishya Kurup, Leila Zabihi Diba, Megan Watson, Holly Ramage, Botond Z. Igyártó

**Affiliations:** 1 Thomas Jefferson University, Department of Microbiology and Immunology, Philadelphia, Pennsylvania, United States of America; 2 Baylor University, Department of Biomedical Studies, Waco, Texas, United States of America; La Jolla Institute for Allergy and Immunology, UNITED STATES

## Abstract

Nucleoside modified mRNA combined with Acuitas Therapeutics’ lipid nanoparticles (LNPs) has been shown to support robust humoral immune responses in many preclinical animal vaccine studies and later in humans with the SARS-CoV-2 vaccination. We recently showed that this platform is highly inflammatory due to the LNPs’ ionizable lipid component. The inflammatory property is key to support the development of potent humoral immune responses. However, the mechanism by which this platform drives T follicular helper (Tfh) cells and humoral immune responses remains unknown. Here we show that lack of Langerhans cells or cDC1s neither significantly affected the induction of PR8 HA and SARS-CoV-2 RBD-specific Tfh cells and humoral immune responses, nor susceptibility towards the lethal challenge of influenza and SARS-CoV-2. However, the combined deletion of these two DC subsets led to a significant decrease in the induction of PR8 HA and SARS-CoV-2 RBD-specific Tfh cell and humoral immune responses. Despite these observed defects, these mice remained protected from lethal influenza and SARS-CoV-2 challenges. We further found that IL-6, unlike neutrophils, was required to generate normal Tfh cells and antibody responses, but not for protection from influenza challenge.

In summary, here we bring evidence that the mRNA-LNP platform can support the induction of protective immune responses in the absence of certain innate immune cells and cytokines.

## Author summary

The mRNA-LNP vaccine platform has gained much attention with the ongoing SARS-CoV-2 pandemic. Millions of people are exposed to these vaccines daily, but their immune mechanism driving the antibody responses and side effects remains unexplored. Therefore, we tested this vaccine platform in the context of influenza and SARS-CoV-2 infections in mouse models that lack specific innate immune cells or cytokines known to play a role in inducing antibody responses. We show that the combined deletion of antigen-presenting innate immune cells and one of the inflammatory cytokines significantly inhibited adaptive immune cell responses triggered by this platform. However, despite the observed defects, the immune responses driven by the mRNA-LNP vaccine were still able to protect the animals from lethal influenza or SARS-CoV-2 challenges. This high degree of redundancy might be associated with the highly inflammatory nature of this platform.

## Introduction

The vaccine platform based on the nucleoside-modified mRNA developed by Karikó and Weissman at the University of Pennsylvania [1,2], combined with proprietary lipid nanoparticles (LNP) of Acuitas Therapeutics [3], gained much attention with the ongoing SARS-CoV-2 pandemic. The platform was previously widely tested in animal models, and the studies reported induction of T follicular helper (Tfh) cells and robust protective antibody responses [4,5]. However, the immune mechanism by which this platform supports adaptive immune responses remains uncharted. With millions of doses of mRNA-LNP-based SARS-CoV-2 vaccines administered daily into humans, dissecting this platform’s immune mechanism and the acute and possible long-term side effects associated with its use are of significant human health interest. The nucleoside-modified and purified mRNAs do not induce strong inflammatory responses [1,2,6]. Still, the ionizable lipid component of these LNPs is highly inflammatory, causes rapid and robust neutrophil infiltration to the injection site, and was responsible for the development of the inflammatory responses characterized by the presence of high levels of inflammatory cytokines, such as IL-1β and IL-6, and chemokines [7]. How the innate inflammatory reactions triggered by these ionizable lipids are translated to adaptive immune responses has not yet been defined. As professional antigen-presenting cells, dendritic cells (DCs) play critical roles in bridging innate and adaptive immune responses [8]. DCs and DC-derived cytokines such as IL-6 are required to initiate the differentiation of naïve CD4^+^ T cells towards the Tfh cell lineage [9]. Whether DC subsets and IL-6 also regulate adaptive immune responses driven by the mRNA-LNP platform, remains to be determined.

Using mice deficient in specific DC subsets, IL-6, or neutrophils, in combination with influenza and SARS-CoV-2 challenge models, here we show that the mRNA-LNP platform can support protective immune responses in the absence of specific DC subsets, IL-6 and neutrophils.

## Results

### LCs and cDC1s show redundancy in driving anti-influenza and anti-SARS-CoV-2 immune responses triggered by the mRNA-LNP vaccine platform

**Fig 1:**
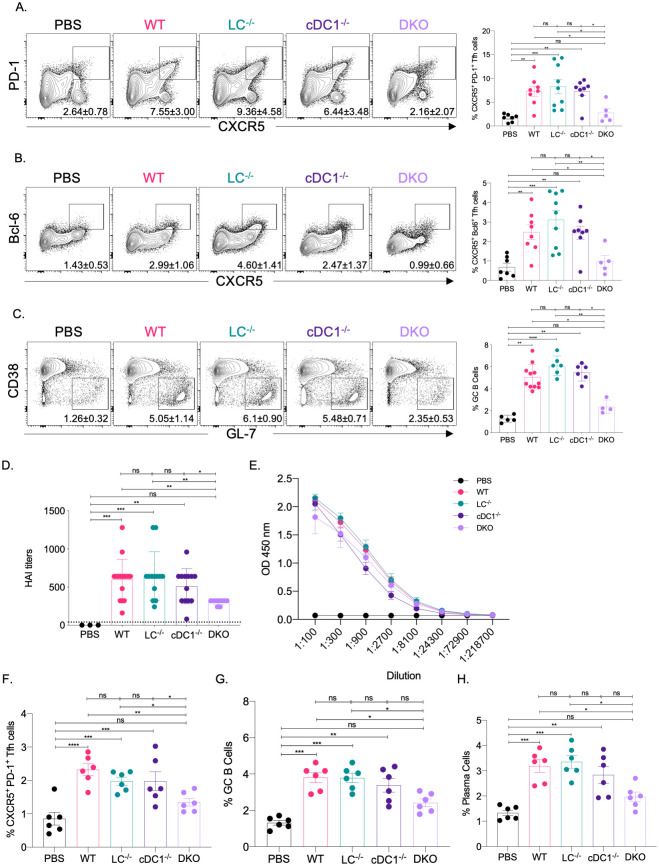
LCs and cDC1s play redundant roles in anti-flu and SARS-CoV-2 adaptive immune responses triggered by the mRNA-LNP platform. **A**. and **B**. The indicated mice were injected with 10 μg of mRNA-LNP coding for PR8 HA and the Tfh cell responses assessed in the skin draining lymph nodes 7 days post immunization. Mice injected with PBS served as control for basal levels. On the left representative flow plots while on the right summary graphs are presented. **C**. As in **A**, except that the lymph nodes were harvested 14 days post immunization and the GC responses determined using flow cytometry. On the summary graphs the % of Tfh cells refers to CD4^+^ T cells in the CD44/CD62L gate, while the % of GC B cells refers to B cells in the B220/CD138 gate (S1 Fig). **D**. and **E**. Serum samples from mice euthanized 14 days post immunization were assessed using HAI and ELISA. **F**. through **H**., as in **A** and **C**, except that the samples for Tfh cell responses were also harvested 14 days post-immunization with mRNA-LNP coding for SARS-CoV-2 RBD. Data were pooled from 2–3 independent experiments. Each dot represents a separate animal.

The mRNA-LNP platform in which nucleoside-modified mRNA is combined with the proprietary LNPs of Acuitas Therapeutics drives effective adaptive immune responses in pre-clinical animal vaccine studies [3,10]. An LNP formulation with a different ionizable lipid from the same company is used in the Pfizer/BioNTech SARS-CoV-2 vaccine [11]. However, very little is known about the immune mechanism by which this platform supports the induction of Tfh cells and humoral immune responses. DCs, including Langerhans cells (LCs) and cDC1s, play essential roles in the induction of Tfh cells [12–16]. Therefore, here we tested the contribution of LCs and cDC1s in regulating adaptive immune responses triggered by this mRNA-LNP platform. Mice deficient in LCs (huLang-DTA, LC^-/-^) [17], cDC1s (Batf3^-/-^, cDC1^-/-^) [18], or both (huLang-DTA by Batf3^-/-^, DKO) [19], and littermate WT controls were intradermally immunized with 10 μg of mRNA-LNP coding for PR8 HA [20] as we previously described [7]. Seven and fourteen days later the antigen-specific Tfh and B cell responses were analyzed by flow cytometer in the skin draining lymph nodes, using the gating strategies presented in S1 Fig. We found that in the absence of LCs or cDC1s, the Tfh and B cell responses specific to PR8 HA were comparable with WT levels (Fig 1A–1C and S2 Fig). However, when both DC subsets were missing, the Tfh and B cell responses were significantly reduced (Fig 1A–1C and S2 Fig). HAI analyses of the serum samples harvested 14 days post-immunization with PR8 HA, corroborated the flow cytometry data and revealed significant decrease in the DKO mice (Fig 1D). However, the total anti-HA serum IgGs determined by ELISA showed no major difference between WT and DC knockouts (Fig 1E).

To determine whether our findings can be generalized to other antigens, we immunized LC^-/-^, cDC1^-/-^, DKO, and littermate WT controls intradermally with 10 μg of mRNA-LNP coding for SARS-CoV-2 RBD [10]. Fourteen days later the skin draining lymph nodes were harvested. The Tfh and B cell responses were characterized using flow cytometry. The flow analyses showed that the RBD-specific Tfh and B cell responses (GC and plasma cells), similarly to the influenza antigen, significantly decreased in the combined absence of LCs and cDC1s (Fig 1F–1H). Thus, LCs and cDC1s play a redundant role in the induction of adaptive immune responses by the mRNA-LNP platform.

### Immune responses induced by the remaining APCs confer protection from lethal viral challenges

**Fig 2:**
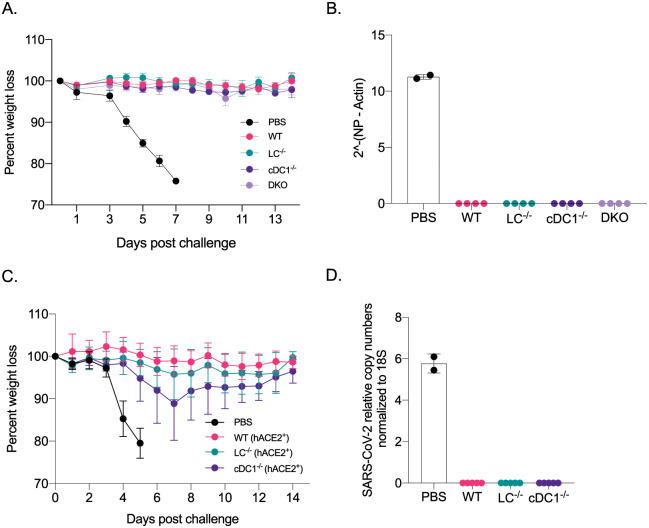
The mRNA-LNP platform supports the development of protective immune responses towards flu and SARS-CoV-2 even in the absence of certain DC subsets. **A**. The indicated animals were immunized with 10 μg of mRNA-LNP coding for PR8 HA or injected with PBS. Fourteen days later the animals were challenged with 5,000 TCDI PR8 influenza virus and the weight drop monitored as presented. Data from two independent experiments pooled, 5–8 mice/group. **B**. Lung samples harvested 7 days post PR8 challenge were assessed using RT-qPCR for viral RNA. **C**. As in **A**, but the indicated mice were immunized using 5 μg of mRNA-LNP coding for SARS-CoV-2 RBD and then 14 days later challenged with 10^5^ PFU SARS-CoV-2. Data from two independent experiments were pooled, 7–10 mice/group. **D**. as in **B**, except the lung samples were tested for the presence of SARS-CoV-2 using qPCR. Each dot represents a separate animal.

Mice lacking LCs, cDC1s, or both, still mounted WT levels of antigen-specific antibodies (Fig 1E). With PR8 HA immunizations, the HAI titers were magnitudes higher (Fig 1D) than the generally accepted protective levels (1:40). To test whether the immune responses generated in the absence of specific DC subsets confer protection against a lethal influenza challenge, we repeated the experiments presented above. On day 14, post-immunization, we challenged the mice with lethal doses of PR8 influenza intranasally. Unimmunized control mice (PBS), consisting of WT and different DC knockouts, dropped weight considerably and were euthanized several days post-challenge (Fig 2A and S3A Fig). No significant differences were observed between the genotypes (Fig 2A and S3A Fig), while all the immunized groups, regardless of DC subset deficiency, remained protected (Fig 2A). The protective immunity was further confirmed by RT-qPCR on lung samples (Fig 2B). Thus, with the mRNA-LNP platform, in the absence of LCs and cDC1s, other APCs can drive protective anti-influenza immune responses.

Next, we sought to determine whether this mRNA-LNP platform can also promote protective immune responses towards SARS-CoV-2 in the absence of LCs and cDC1s. For the SARS-CoV-2 challenge studies, we first bred the huLang-DTA [17] and Batf3^-/-^ [18] mice to hACE-2 transgenic mice [21]. The generated genotypes were confirmed using PCR, and the hACE-2 positive and negative mice were further tested for virus susceptibility (S3B Fig). The optimal SARS-CoV-2 dose was determined in house (S4 Fig). The resulting mice were then intradermally injected with 5 μg of mRNA-LNP coding for SARS-CoV-2 RBD or PBS as presented above. On day 14, post-injection, we challenged the mice with a lethal dose of SARS-CoV-2 intranasally. WT mice not carrying the human ACE-2 receptor were resistant to the infection (S3B Fig). PBS injected hACE-2 mice, and different hACE-2 DC knockouts dropped weight considerably and were euthanized several days post-challenge (S3B Fig). No significant differences were observed between the genotypes (S3B Fig), while all the immunized groups, regardless of DC subset deficiency, remained protected (Fig 2C). The protective immunity was further confirmed by RT-qPCR on lung samples (Fig 2D). Thus, the mRNA-LNP platform can drive protective anti-SARS-CoV-2 immune responses in the absence of LCs and cDC1s.

### IL-6 is required for optimal induction of Tfh and B cell responses

**Fig 3:**
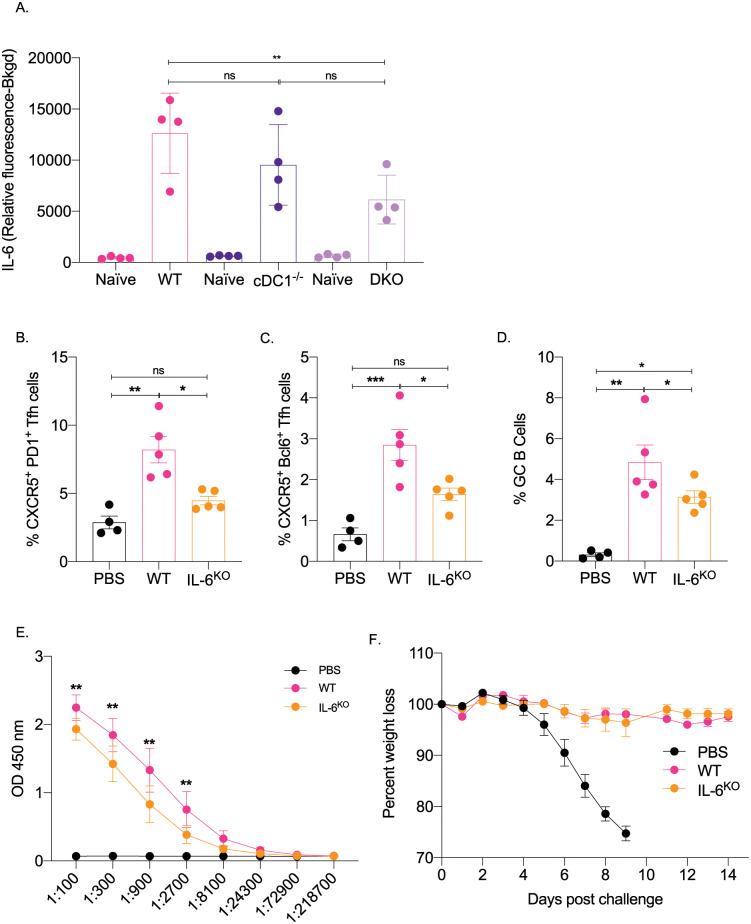
IL-6 is required for the induction of adaptive immune responses by the mRNA-LNP platform. **A**. The indicated mice were injected intradermally with 2.5 μg of non-coding polycytosine mRNA-LNP and the skin samples tested for IL-6 with Luminex 24 hours later. **B**. and **C**. WT and IL-6 knockout mice were intradermally injected with 10 μg of mRNA-LNP coding for PR8 HA or PBS. Seven days later the Tfh cells were characterized in skin draining lymph nodes using flow cytometry. **D**. as in **B** and **C**, except the lymph nodes were harvested 14 days post injection and the GC B cell responses determined by flow cytometer. Each dot represents a separate animal. **E**. Serum samples at day 14 post injection were assessed for anti-HA IgG levels using ELISA. **F**. The indicated animals were immunized with 10 μg of mRNA-LNP coding for PR8 HA or injected with PBS. Fourteen days later the animals were challenged with 5,000 TCDI PR8 influenza virus and the weight drop monitored as presented. Data from two independent experiments pooled, 5–9 mice/group.

IL-6 in mice in inflammatory settings is required for the induction of Tfh cells [22,23] and functions as a B cell growth factor [24]. We found that this mRNA-LNP platform induces high levels of IL-6 at the injection site [7], which is decreased in the DKO mice (Fig 3A). Therefore, we tested whether IL-6 plays a role in adaptive immune responses triggered by the mRNA-LNP platform. Age-matched IL-6 knockout and WT mice were immunized intradermally with 10 μg of PR8 HA mRNA-LNP. The Tfh cells and the B cell responses were assessed by flow cytometry seven- and fourteen-days post-immunization, respectively. We found that in the absence of IL-6, both Tfh cell induction and B cell responses were significantly affected (Fig 3B–3D). Immunized IL-6 deficient mice also showed decreased anti-HA serum IgG levels (Fig 3E). To determine whether the mounted immune responses in the absence of IL-6 can confer protection from subsequent exposure to influenza, we immunized WT and IL-6 knockout mice with 10 μg of PR8 HA mRNA-LNP and 14 days later exposed them to a lethal dose of PR8 influenza virus. We found that despite the reduced adaptive immune responses, the IL-6 deficient mice remained protected (Fig 3F). Thus, IL-6, similarly to other inflammatory models, also plays a critical role in driving Tfh and B cell responses by the mRNA-LNP platform. However, the blunted immune responses induced by this vaccine platform in the absence of IL-6 can still confer protection from lethal viral challenge.

### Neutrophils are dispensable for the Tfh and B cell responses induced by the mRNA-LNP platform

The mRNA-LNP platform through its ionizable lipid component triggers robust, rapid, but transient neutrophil infiltration at the delivery site [7]. Through induction of inflammatory milieu and activation of local APCs, and other cellular and molecular interactions, neutrophils can contribute to the generation of downstream adaptive immune responses [25–29]. Therefore, to test the contribution of the neutrophils in regulation of adaptive immune responses triggered by the mRNA-LNP, we depleted them two days before immunization using 1A8 (anti-Ly-6G) antibody [30]. Control mice were injected with isotype antibodies. The absence of neutrophils at the time of immunization was confirmed using flow cytometry on blood cells (S5A and S5B Fig). The mice were then immunized with 10 μg of PR8 HA mRNA-LNP as described above. Seven and fourteen days later, the Tfh cells and B cell responses, respectively, were assessed in the skin draining lymph nodes using flow cytometry. The absence of neutrophils did not significantly affect either PR8 HA-specific Tfh cell development or B cell responses (S5C–S5E Fig). The serum anti-HA IgG levels showed slight decrease in the absence of neutrophils, which however, did not reach significance (S5F Fig). Furthermore, neutrophil depleted and PR8 HA mRNA-LNP immunized animals showed slight but not significant weight drop upon challenge with a lethal dose of influenza (S5G Fig). Thus, these data suggest that neutrophils are unlikely to play a critical role in regulating adaptive immune responses induced by the mRNA-LNP platform.

## Discussion

Here we show that the mRNA-LNP platform widely used in preclinical animal vaccine studies promotes protective immune responses against influenza and SARS-CoV-2 infections in the absence of LCs and cDC1s. We further identified IL-6 as an important inflammatory cytokine in supporting the induction of adaptive immune responses by this platform and showed that neutrophils were dispensable for these responses.

We recently described that the mRNA-LNP platform built on the nucleoside modified mRNA pioneered at the University of Pennsylvania by Karikó and Weissman [1,2] combined with the proprietary LNPs of Acuitas Therapeutics is highly inflammatory independently of the route of administration [7]. The inflammatory property of this platform was due to its ionizable lipid component that led to activation of multiple inflammatory pathways and the production of a variety of different inflammatory cytokines and chemokines [7]. The inflammatory nature of the LNPs is crucial for the induction of adaptive immune responses, but no detectable Tfh and B cell responses are mounted against the LNP components after the first exposure (S6 Fig). The mRNA delivered alone also does not induce adaptive immune responses (S7 Fig). However, it is unknown how the innate inflammatory responses induced by the LNPs support the adaptive immune responses, especially Tfh cell and B cell responses. DCs play a critical role in bridging the innate and the adaptive immune responses [8]. We found that with the mRNA-LNP platform, LCs and cDC1s play redundant roles in driving Tfh cells and antibody responses. In certain inflammatory conditions, unlike the LC^-/-^ mice, the Batf3 mice have been reported to have compensatory cDC1 development [31,32]. The compensatory cDC1 development could potentially explain the normal adaptive immune responses and protection from viral challenge reported here with the Batf3 KO mice. However, we noted no compensatory repopulation of the migratory cDC1s with the mRNA-LNP platform (S8 Fig). Thus, these data support that the lack of change in adaptive immune responses in the Batf3 KO mice are not due to compensatory effects of DCs. In support of this, the combined deletion of LCs and cDC1s led to a significant decrease in the adaptive immune responses, confirming their essential roles in driving Tfh cells and B cell responses. However, even in the absence of these cell types, the mRNA-LNP platform induced antibody responses of WT levels and the immune responses mounted could confer protection from subsequent viral challenges. While the antibody titers showed no difference, the DKO-derived serum was less effective in an HAI assay. This suggests that the antibodies or some of the antibodies formed against HA in the absence of LCs and cDC1s might not effectively prevent agglutination but could still contribute to the protection, possibly by mediating ADCC [33]. Since the Tfh cell formation is severely affected in the DKO mice, it is possible that Th1 cells previously reported in the influenza settings [34] could provide the necessary help for the B cell responses, which here might be extrafollicular in nature. The antibody responses in this case, are likely driven by the cDC2s that are also potent in supporting antibody responses [12]. The involvement of the cDC2s in driving the adaptive immune responses is also indirectly supported by our preliminary observation that upon intradermal immunization with DiI-labeled LNP complexed with mRNA coding for GFP the cDC2s were heavily targeted (manuscript in preparation). Since the platform is highly inflammatory, and the lipid-based nature of this platform allows the mRNA to reach and transduce cells far from the injection site, it is likely that lymph node resident APCs could contribute to antigen presentation and regulation of adaptive immune responses. Antigen presentation by B cells is needed for the final maturation of Tfh cells [35]. B cells in the lymph node might also get transduced by the mRNA or have access to secreted antigens premade by other cells or presented to them by DCs. However, the details of the cellular and molecular interactions triggered by this platform remain to be elucidated. Interestingly, despite that the intramuscularly delivered mRNA-LNP vaccine can reach almost any organ in the body [36], we observed that the detectable immune responses were limited to the LNs draining the area of injection. The four spots vaccination strategy used here targets all the six main skin draining LNs. However, inoculation in one of those four spots, induced immune responses in just the local LN and not in other LNs (S9 Fig). Which strategy induces more robust adaptive immune responses and how delivering in multiple spots affects boosting remain to be determined. In humans, the vaccines are often delivered in the same area, including booster shots, and whether splitting the dose in multiple locations would be beneficial should probably be explored.

Tfh cells and B cell responses in mice require the presence of IL-6 [22,23]. DCs are thought to be the source of IL-6 [37], and IL-6 is needed to initiate the Tfh cell program, including upregulation of the signature transcription factor Bcl-6 and CXCR5 [22,38]. Our data support that IL-6 is also required for the induction of adaptive immune responses with this platform. However, further studies will be needed to identify the cellular source of IL-6. We observed high levels of IL-6 at the site of injection [7], which decreased in DKO mice, supporting its DC origin or DC dependence. Still, cytokines present at the interface of the immunological synapse between APCs and naïve T cells are likely to be of higher importance [39,40]. The absence of IL-6 did not lead to a complete lack of Tfh cells and antibody responses. Therefore, other cytokines and co-stimulatory molecules previously described to play essential roles in regulating humoral immune responses, such as IL-1β and IFNα, are expected to contribute [9,35]. At the injection site, we observed high levels of IL-1β and an increase in the *NLRP3* gene transcript levels [7]. A bone-marrow-derived macrophage cell line, exposed *in vitro* to LNP induced dose-dependent IL-1β secretion that was reliant on the presence of Caspase-1/11 (S10 Fig). Whether, in this case, the IL-1β production is the consequence of canonical or non-canonical inflammasome activation and plays role in the induction of antibody responses remains to be explored [41].

The immunological roles of DC subsets in SARS-CoV-2 infections are incompletely understood. Recent human studies have indicated that SARS-CoV-2 infections can affect DC biology [42–45]. We found that naïve mice that lacked LCs or cDC1s and littermate WT controls succumbed to intranasal SARS-CoV-2 infections at the same rate (S3B Fig). These data suggest that the absence of these DC subsets at the time of exposure does not render the host immunocompromised. More importantly, our vaccination studies showed a high degree of redundancy between DC subsets and cytokines. This vaccine platform can still induce protective responses even in the absence of specific antigen-presenting cells, neutrophils, and inflammatory cytokines. These are highly relevant findings and provide hope to individuals who might lack certain DC subsets or otherwise be immunocompromised to develop protective immune responses [46].

The mRNA-LNP platform is highly inflammatory and activates distinct inflammatory pathways and cells [7]. Thus, our findings that the mRNA-LNP vaccine can drive protective immune responses in the absence of certain innate immune cells and cytokines even at low dose (S11 Fig), are not surprising. While this might be a good thing from the SARS-CoV-2 vaccine’s perspective, we should tread carefully. Local, such as so-called Covid arm [47], and distal inflammatory reactions in the heart and brain have been reported with the mRNA-LNP platform [48–53]. The robust inflammation driven by the ionizable lipid component of the LNPs [7] likely contributed to the innate and adaptive immune reprogramming recently reported with this vaccine platform [54]. Thus, we think that more basic and translational research would be needed before this platform gets full approval for human use.

## Materials and methods

### Ethics statement

Institutional Care and Use Committee at Thomas Jefferson University approved all mouse protocols. Protocol number: 02315.

### Mice

HuLang-DTA [17], Batf3^-/-^ [18], huLang-DTA by Batf3^-/-^ [19] mice were previously published and bred in house. WT C57BL/6J, IL-6 KO and huACE-2 mice [21] were purchased from Jax and bred in house. HuLang-DTA and Batf3^-/-^ mice were bred to huACE-2 mice in house. All experiments were performed with 6–15 weeks old female and male mice. Mice were housed in microisolator cages and fed autoclaved food.

### mRNA-LNPs

For our studies, we used an LNP formulation proprietary to Acuitas Therapeutics described in US patent US10,221,127. These LNPs were previously carefully characterized and widely tested in preclinical vaccine studies in combination with nucleoside-modified mRNAs [3,4,10,20]. The following, previously published mRNA-LNP formulations were used: PR8 HA mRNA-LNP [20] and SARS-CoV-2 RBD mRNA-LNP [10].

### Cells and viruses

A/Puerto Rico/8/1934 influenza stock was a generous gift from Dr. Scott Hensley (University of Pennsylvania). Vero E6 and Vero CCL81 were obtained from ATCC and were cultured in 1X DMEM (Corning, Cat# 10-013-CV), supplemented with 5% (v/v) fetal bovine serum (FBS), 1% (v/v) penicillin/streptomycin, and were maintained at 37 °C and 5% CO2. SARS-CoV-2 was obtained from BEI (WA-1 strain). Stocks were prepared by infection of Vero E6 cells in 2% serum for five days, freeze-thawed, and clarified by centrifugation (PO). Titer of stock was determined by plaque assay using Vero E6 cells and were 1x10^7^ pfu/mL and 1.5x10^6^ TCID 50/mL. This seed stock was amplified in Vero CCL81 (P1) at 1.5x10^6^ TCID 50/mL. All work with infectious virus was performed in a Biosafety Level 3 laboratory and approved by the Institutional Biosafety Committee and Environmental Health and Safety.

### SARS-CoV-2 antibody

SARS-COV/COV-2 S1 human monoclonal antibody obtained from Absolute Antibody (AB01680-10.0).

### Virus titration

To determine infectious titers ten-fold serial dilutions of filtered supernatants were prepared in DMEM containing 10% FBS and added to Vero CCL81 cells in a 96-well plate. Cells were incubated under standard cell culture conditions at 37 °C and 5% CO2 for 48 h. Cells were fixed in 4% formaldehyde/PBS for 15 min at room temperature and then washed three times with PBST. Cells were blocked (2% BSA/PBST) for 60 min and incubated in primary antibody directed against the S1 domain of the human SARS-CoV-2 Spike protein overnight at 4 °C. Cells were washed 3x in PBST and incubated in secondary antibody (Goat anti-human IgG Alexa 488, ThermoFisher, and Hoechst 33342 ThermoFisher) for 2 h.

### Intradermal immunization

Intradermal immunizations were performed as we previously described [7]. Briefly, the day before injections, the hair from the back skin of the mice was removed using an electric clipper, and then the site of injections wet-shaved using Personna razor blades. The next day the mice were injected intradermally with 2.5 μg/spot mRNA-LNP in 20 μl PBS (4 spots, 10 μg total) or PBS.

### Characterization of Tfh and B cell responses

At day 7 (peak of T cell responses; [55]) and 14 post-injections (peak of B cell responses; [56]), the mice were sacrificed and the skin draining lymph nodes (axillary, brachial and inguinal) harvested. Single-cell suspensions were generated using mechanical disruption through cell strainers. The cells were stained with either Tfh cell or B cell panels. The Tfh cell panel contained: fixable viability dye (Thermo Fisher), CD4 (GK1.5), CD44 (IM7), CD45 (OX-7), CD62L (H1.2F3), CD69 (H1.2F3), CXCR5 (L138D7), PD-1 (29F.1A12) and Bcl-6 (K112-91) from BioLegend and BD Biosciences. The gating strategy can be found in S1A Fig. The B cell panel consist of dump (fixable viability dye, F4/80, CD11b), CD38 (90), B220 (RA3-6B2), CD138 (281–2), GL-7 (GL-7), Sca-1 (D7), IgD (11-26c.2a) and IgM (RMM-1). The gating strategy is presented in S1B Fig. The stained samples were run on LSRFortessa (BD Biosciences) and the resulting data analyzed with FlowJo 10.

### Intranasal challenge with PR8 influenza and SARS-CoV-2

The influenza dose used in these studies was previously described [20,57], while the SARS-CoV-2 dose was determined in house using hACE-2 mice (S4 Fig). Mice were anesthetized by intraperitoneal injection with a mixture of Xylazine/Ketamine and inoculated intranasally as follows. Mice received 5000 TCDI PR8 influenza virus or 10^5^ PFU SARS-CoV-2 (WA-1 strain). Subsequently the mice were monitored daily for distress and weight loss. The weight loss data are presented as percent of original body weight.

### Viral RNA quantification

Mouse lungs from influenza challenged mice were collected, and flash frozen prior to storage at -80 °C. RNA was prepared using the RNeasy Micro kit (Qiagen, Cat: 74004), following the manufacturer’s instructions. 168 ng of RNA was reverse-transcribed using iScript Reverse Transcription Supermix for RT-PCR (Bio Rad, Cat: 1708840), following the manufacturer’s instructions. Quantitative PCR was performed with iTaq Universal SYBR Green Supermix (Bio Rad, Cat: 1725121), following the manufacturer’s instructions. Relative viral load was measured by ΔCT of PR8 influenza virus nucleoprotein (NP) using mouse β-Actin as a reference gene. Forward (5’) Pr8 NP: CAGCCTAATCAGACCAAATG, Reverse (3’): TACCTGCTTCTCAGTTCAAG. Forward (5’) β-Actin AGATTACTGCTCTGGCTCCTAGC and Reverse (3’): ACTCATCGTACTCCTGCTTGCT [58,59]. For the SARS-CoV-2 challenge studies portions of lungs from infected animals were weighed and placed in Trizol. Lung samples were homogenized using Omni beads (1.4 mm ceramic: Part no 19–627) in a Bead Ruptor 12 (Omni International). 100 μl of the lung homogenate was transferred to a new tube containing 900 μl Trizol and RNA was extracted using the Zymo Clean and Concentrator 25 kit according to the manufacturer’s instructions. To quantify viral RNA, complimentary DNA (cDNA) was synthesized using 1 μg of input RNA with random hexamer primers (Life Technologies) with MMLV reverse transcriptase (Invitrogen) in a total volume of 20 μl. cDNA reactions were diluted 1:5 and 20 μl of each diluted sample was used to make a pooled reference. The pooled reference was used for subsequent 10-fold dilutions to generate a standard curve for all targets being measured. cDNA reactions were further diluted 1:5 (1:25 total dilution) and SYBR green reactions contained 5 μl of 2x Maxima SYBR green/Rox qPCR Master Mix (Thermo), 5 μl of diluted cDNA, 5 pmol of both forward and reverse primers, analyzed by qPCR and the relative abundance of each target was calculated using the standard curve. The relative values for viral RNA were normalized to a control RNA (18S rRNA) and compared between experimental conditions. The following primers were used:

SARS-CoV-2 N Forward: TTACAAACATTGGCCGCAAA, SARS-CoV-2 N Reverse: GCGCGACATTCCGAAGAA, 18S Forward: AACCCGTTGAACCCCATT, 18S Reverse: CCATCCAATCGGTAGTAGCG.

### ELISA

Nunc Immuno 96 well plates (Fisher Scientific) were coated with 1μg/ml (50 μl/well) HA protein (Sino Biological) diluted in carbonate/bicarbonate buffer (Fisher Scientific) overnight at 4 °C or 1 hour at 37 °C. After washing and blocking with TBS for 1 hour the serum samples were diluted and added to the plate. Serially diluted HA-specific monoclonal antibody (Sino Biological) served as standard. Anti-mouse IgG-HRP (1:20,000; Fisher Scientific) in combination with TMB (Fisher Scientific) solution was used for detection. The signals were read at 450 nm using accuSkan FC microplate photometer (Fisher Scientific).

### HAI

The assay was performed as previously described [20]. Briefly, the sera were heat inactivated. Titrations were performed in 96-well, round-bottom plates (BD Biosciences). Sera were serially diluted twofold and added to four agglutinating doses of virus in a total volume of 100 μl. Turkey erythrocytes (Lampire Biological Laboratories) were added (12.5 μl of a 2% [vol/vol] solution). The erythrocytes were gently mixed with sera and virus, and agglutination was read after incubating for 1 h at room temperature. HAI titers were expressed as the inverse of the highest dilution that inhibited four agglutinating doses of influenza virus.

### Neutrophil depletion

Neutrophils were depleted using published protocol [30]. Briefly, mice were either injected with 1 mg 1A8 recognizing Ly-6G antigen (BioLegend) or isotype control antibodies (clone: RTK2758; BioLegend) intraperitoneally two days before immunization. Depletion efficiency was monitored in blood using flow cytometry.

### Luminex for IL-6

WT, cDC1^-/-^ and DKO mice were injected intradermally with 2.5 μg/spot mRNA-LNP in 20 μl PBS or PBS. Luminex on injected skin samples were performed as we previously described [7].

### *In vitro* assay for IL-1β secretion

Bone marrow-derived macrophages were harvested from WT C57BL/6J mice or Caspase 1/11^-/-^ mice of the same genetic background and immortalized as described in Rodrigue-Gervais et. al [60]. Cells were cultured at 37 °C 5% CO_2_ in RPMI-1640 medium containing 10% heat-inactivated newborn calf serum, 2 mM glutamine, 100 μg/ml penicillin/streptomycin, 0.01 M HEPES, 100 mM sodium pyruvate, and 33 μM 2-mercaptoethanol. Cells were then plated at a concentration of 1 x 10^6^ cells per well in media containing 50 ng/mL of LPS and left for 4 hours. After LPS pre-treatment, plates were spun down and the media partially removed. Media containing LNPs, nigericin, or vehicle were added. Supernatant was collected 24 hours and IL-1β concentration was measured using DuoSet ELISA kits (R&D systems) according to manufacturer’s instructions.

### Statistical analyses

All data were analyzed with GraphPad Prism version 9.0.0. The data sets were first analyzed for normal Gaussian distribution using Shapiro-Wilk test and based on the outcome either one-way ANOVA (normal) or Kruskal-Wallis (non-normal) tests were used to compare the different groups. P<0.05 was considered significant. ns = not significant, *p<0.05, **p<0.005, ***p<0.001.

## Supporting information

S1 Fig Gating strategy for identification of Tfh cells, GC B cells, and plasma cells. (EPS)

S2 Fig Total cell numbers. (EPS)

S3 Fig The absence of DC subsets at the time of exposure does not render the host immunocompromised. **A**. The indicated naïve animals were challenged with 5,000 TCDI PR8 influenza virus and the weight drop monitored as presented. Data from two independent experiments pooled, minimum 5 mice/group. **B**. As in **A**, but naïve animals were exposed to 10^4^ PFU of SARS-CoV-2 and the weight drop monitored as presented. One representative experiment is shown with 5 mice/group.
(EPS)

S4 Fig SARS-CoV-2 dosing. hACE2^+^ mice were inoculated intranasally with increasing doses of SARS-CoV-2 and weight drop recorded as presented. One representative experiment is shown with multiple mice.
(EPS)

S5 Fig Lack of neutrophils at the time of immunization does not affect the induction of adaptive immune responses by the mRNA-LNP platform. **A**. and **B**. WT mice were either injected with isotype or neutrophil depleting antibodies targeting Ly-6G antigen (1A8) two days before immunization. **A**. Representative flow plot showing complete neutrophil depletion in the blood of 1A8 antibody injected mouse. **B**. Summary graph with multiple mice. Naïve indicates pre-injection levels of the neutrophils for the same mice. **C**. and **D**. Mice from **B** were intradermally injected with 10 μg of mRNA-LNP coding for PR8 HA or PBS. Seven days later the Tfh cells were characterized in skin draining lymph nodes using flow cytometry. **E**. as in **C** and **D**, except the lymph nodes were harvested 14 days post injection and the GC B cell responses determined by flow cytometer. Each dot represents a separate animal. **F**. Serum samples at day 14 post injection were assessed for anti-HA IgG levels using ELISA. **G**. The indicated animals were immunized with 10 μg of mRNA-LNP coding for PR8 HA or injected with PBS. Fourteen days later the animals were challenged with 5,000 TCDI PR8 influenza virus and the weight drop monitored as presented. Data from two independent experiments pooled, 6 mice/group.
(EPS)

S6 Fig No detectable Tfh and B cell responses are mounted against empty LNP components. cDC1^-/-^ mice were exposed to PBS, empty LNP or mRNA-LNP coding for PR8 HA. Seven days later the Tfh and B cell responses were characterized using flow cytometer. One experiment with multiple mice is shown.
(EPS)

S7 Fig mRNA-LNP dose titration experiment. **A**. and **B**. WT animals were injected with increasing doses of mRNA-LNP coding for PR8 HA, or PBS or 10 μg of mRNA. Fourteen days later the B cell responses in the skin draining lymph nodes were determined using flow cytometry. **C**. Serum samples from **A** were assessed using HAI. Dotted line marks the 1:40 titer. Data from two independent experiments were pooled. Each dot represents a separate animal.
(EPS)

S8 Fig Exposure to mRNA-LNP does not induce compensatory development of migratory cDC1s. cDC1^-/-^ mice were exposed to PBS, empty LNP or mRNA-LNP coding for PR8 HA. Seven days later the % of cDC1s in the skin draining lymph nodes were determined using flow cytometer. One experiment with multiple mice is shown.
(EPS)

S9 Fig The adaptive immune responses are limited to the site of injection. WT mice were injected in one spot with PBS or mRNA-LNP coding for PR8 HA. Fourteen days later the lymph nodes were harvested from the non-immunized side (nis) or the immunized side (is) and the % of GC B cells determined using flow cytometer. One experiment with multiple mice is shown.
(EPS)

S10 Fig Macrophages respond with dose and caspase 1/11-dependent IL-1β production upon exposure to empty LNPs. WT and Caspase 1/11 deficient macrophages were exposed to an increasing amount of empty LNPs for 24 hours, and then the secreted IL-1β measured in the supernatant using ELISA. Data from multiple experiments were pooled.
(EPS)

S11 Fig Lower dose of mRNA-LNP still induces protective adaptive immune responses. **A**. The indicated animals were immunized with 1 μg of mRNA-LNP coding for PR8 HA or injected with PBS. Fourteen days later the animals were challenged with 5,000 TCDI PR8 influenza virus and the weight drop monitored as presented. Data from two independent experiments pooled, minimum 5 mice/group.
(EPS)
